# Insights on Recruitment, Implementation, and Movement Pattern Detection by Exploring the Feasibility of Sensor-Based Insole Technology in Long-Term Care: Mixed Methods Feasibility Study

**DOI:** 10.2196/83133

**Published:** 2026-02-18

**Authors:** Alexa von Bosse, Michael Ziegler, Steffen Heinrich

**Affiliations:** 1 School of Health Sciences, Dementia Competence Centre, IPW Institute of Applied Nursing Science, Eastern Switzerland University of Applied Sciences, Vadianstrasse 29, St Gallen, 9000, Switzerland, 41 58 257 14 9; 2 School of Management, IPM Institute for Information and Process Management, Eastern Switzerland University of Applied Sciences, St Gallen, Switzerland

**Keywords:** sensor-based monitoring, wearable technology, gait analysis, agitation detection, ambient-assisted living, feasibility study, technology implementation, long-term care, nursing home

## Abstract

**Background:**

Sensor-based footwear is increasingly discussed as a promising tool for mobility monitoring and fall-risk assessment, yet its applicability in long-term care remains largely unexamined. In particular, little is known about whether such systems and the study procedures needed to evaluate them can be feasibly integrated into dementia care settings. This feasibility study provides practice-based evidence of using a sensor-equipped insole system with cognitively impaired residents.

**Objective:**

The study examines recruitment feasibility, integration of the device into daily care routines, and the operationalization of the study design under everyday institutional conditions. Furthermore, it explores whether movement patterns, including agitation-related behaviors, can be detected in situ, acknowledging the exploratory nature of this aim due to the rarity and unpredictability of natural agitation episodes.

**Methods:**

An exploratory feasibility study was conducted in 2 long-term care facilities in Eastern Switzerland. Six residents with mild-to-moderate cognitive impairment and increased fall risk were recruited through a multistep, staff-supported process, offering rare documentation of real-world recruitment constraints. The insole system recorded continuous gait and movement data during daily activities and weekly walking tests. Controlled simulations of agitation-related patterns were conducted to generate reference data, as natural agitation events were infrequent and difficult to capture systematically. Feasibility outcomes were informed focusing on recruitment capability, acceptability and adherence, feasibility of continuous data acquisition, and the practicality of integrating the system into daily care routines.

**Results:**

The study identified substantial feasibility constraints, including contextual limitations posed by sedation practices, fluctuating health status, and consent-related barriers. Practical challenges such as inconsistent battery charging, communication gaps across rotating staff, and difficulties achieving adequate shoe fit directly affected protocol adherence and data yield. Continuous sensor-based data collection was technically feasible when the shoes were worn, and machine-learning models consistently classified predefined agitation-related patterns under controlled conditions. Natural agitation episodes were rare, making simulated reference data essential for establishing fundamental detectability. Resident acceptance varied, with some individuals reporting increased perceived stability due to the shoe’s firm construction rather than its vibration feature.

**Conclusions:**

This feasibility study demonstrates that sensor-based footwear is technically feasible and shows situational acceptability in long-term care, while also highlighting key contextual constraints related to recruitment, workflow integration, device handling, and adherence. The findings provide early empirical evidence on what is practically achievable in this population and setting, clarifying the exploratory nature and methodological necessity of using simulated agitation patterns, and underscoring the need for context-sensitive, participatory strategies when introducing sensor-based technologies into dementia care.

## Introduction

### Background

Mobility limitations, gait instability, and a high risk of falls are among the most pervasive challenges in long-term care. Older adults, particularly those living with cognitive impairment, experience fluctuating functional capacities, reduced hazard awareness, and difficulties communicating emerging physical changes [1]. Recent evidence highlights that falls are highly prevalent in long-term care facilities and result from a complex interplay of mobility limitations, cognitive impairment, environmental factors, and organizational conditions. Older adults in long-term care experience markedly higher fall rates than community-dwelling older adults, underscoring the need for reliable approaches to monitor mobility and fall risk in these settings [2]. This elevated fall risk is even more pronounced among residents with dementia, as cognitive impairment further reduces hazard awareness, affects judgment, and limits the ability to respond adequately to environmental challenges [3]. Furthermore, up to 86% of long-term care residents with dementia exhibit agitation, and about 40% experience clinically significant symptoms [4]. These symptoms are clinically relevant not only because of their association with caregiver burden and reduced well-being but also because they can serve as prognostic indicators of disease progression [5].

Despite the clinical relevance of mobility changes and fall risk, everyday monitoring in long-term care largely relies on brief, subjective staff observations conducted under considerable workload pressures [6]. This lack of continuous, objective monitoring restricts the ability of care teams to detect emerging restlessness, identify early indicators of fall risk, or differentiate transient fluctuations from clinically meaningful deterioration. Technological approaches such as multimodal wearables and artificial intelligence (AI)-based detection systems may help address this monitoring gap; however, these systems have rarely been evaluated under real-life long-term care conditions [7], and their usability and acceptance among people with dementia remain insufficiently understood. This gap underscores the need to first evaluate whether such systems can be feasibly implemented in everyday care routines before considering their clinical use.

Usability and acceptance are particularly critical in populations with cognitive impairment, where device comfort, unobtrusive design, intuitive handling, and minimal maintenance demands strongly influence adherence and sustainable use [8]. Trust, perceived usefulness, and low-threshold interaction design further shape acceptance, especially in settings where residents may be unfamiliar with or sensitive to digital technologies [9-11]. Many commercially available wearables, including smartwatches, are perceived as inaccurate in slow walkers, require frequent charging, or are easily misplaced, which limits their suitability for older adults with dementia [12]. These findings underscore the need for context-sensitive and user-centered design principles when deploying sensor-based monitoring approaches in long-term care environments [13].

Against this backdrop, sensor-equipped footwear represents a promising alternative. By embedding sensors directly into everyday shoes, mobility and gait-related parameters can be captured unobtrusively and with minimal behavioral change, reducing the risk of device loss and improving usability [14,15]. Advances in AI further enable the detection of clinically relevant movement patterns, including those associated with agitation or fall risk [16]. Nevertheless, such systems have not yet been systematically evaluated in real-world long-term care settings, and evidence on their feasibility, acceptance, and integration into routine dementia care remains sparse.

In response to these gaps, this study examines the feasibility of using a sensor-based insole system in the everyday context of long-term care. While the system also allows exploratory examination of specific movement characteristics, such as restlessness or agitation-related patterns, its primary purpose in this pilot study is to determine whether such a technology and its associated study procedures can be realistically implemented under the complex and variable conditions of long-term care. Recent research emphasizes the importance of patient-centeredness, outcome orientation, and the acceptability of technologies in vulnerable populations and complex care environments [17,18]. Despite promising technological advances, no established solutions currently use in-shoe sensors to monitor agitation-related motor activity in long-term care, underscoring the exploratory and innovative character of this feasibility study.

### Study Objectives and Research Questions

The aim of this feasibility study is to examine the practicality of introducing a sensor-based insole system into everyday long-term care. Specifically, the study investigates (1) the wearability, usability, and user acceptance among residents with cognitive impairment; (2) the feasibility of integrating the system into routine care workflows, including adherence and compatibility with daily procedures; and (3) the technical feasibility of continuously recording movement patterns and obtaining initial indications of the detectability of health-relevant behaviors, including agitation-related motor activity, under real-world conditions.

Accordingly, the primary objective is to determine whether the system can be realistically used in daily care practice, whether continuous valid movement data can be acquired, and whether exploratory analyses can provide preliminary insights into the detection of abnormal movement patterns associated with increased health risks. Particular attention is given to movement patterns potentially indicative of agitated motor behavior. The vibration component is conceptualized as an adjunctive feature rather than a central intervention under investigation.

The study uses a sensor-equipped insole system that continuously records movement data and applies exploratory AI-based analyses to quantify mobility parameters and identify predefined movement signatures. Beyond monitoring, the system includes an optional vibration feature that delivers low-intensity tactile impulses to the plantar surface. Prior research suggests that such stimulation may enhance sensory feedback and support gait and balance in older adults [19,20], although evidence remains preliminary. Consistent with the feasibility focus, this feature is examined only in an exploratory manner.

Building on these aims and system characteristics, the study was structured around three feasibility domains that align with established objectives for feasibility research. These domains guided the formulation of the research questions (RQs) as outlined as follows:

- Process feasibility (recruitment, study design, and implementation)
  - RQ1: To what extent is the study design including recruitment processes, organizational integration, and day-to-day implementation feasible in long-term care facilities?

- Acceptability and perceived value
  - RQ2: How do residents, particularly those with cognitive impairments, perceive and accept the sensor-based system in daily care?
- Technical feasibility (data capture and system functionality)
  - RQ3: Under real-world conditions, to what extent is it feasible to obtain analyzable movement data during naturally occurring agitation episodes, and which sensor signals show preliminary indicators of potential usefulness for subsequent model development?
  - RQ4: How practical, robust, and reliable is the insole-based sensor system in capturing movement patterns under real-world long-term care conditions?
  - RQ5: Under controlled conditions, to what extent can agitation-related movement patterns be exploratively characterized, and which sensor modalities and machine-learning approaches appear technically feasible for this purpose?

## Methods

### Study Design

This study was designed as an exploratory feasibility study, structured around 3 core feasibility dimensions: process feasibility, acceptability and perceived value, and technical feasibility.

### Sample and Recruitment

Participants were recruited from 2 long-term care facilities in eastern Switzerland, comprising specialized dementia units, general nursing wards, and a unit for residents with comorbid substance use disorders. Eligible participants were residents with mild-to-moderate cognitive impairment, documented increased fall risk and/or notable movement behaviors (eg, agitation), and the capacity to provide informed consent.

Recruitment followed a multistage, staff-supported process. After institutional approval, nursing staff and the research team identified potentially eligible residents and provided individualized verbal and written information about the study’s purpose and procedures. Interested residents were approached personally and invited to participate voluntarily. Sociodemographic data and cognitive status (Mini-Mental State Examination) were collected at baseline.

### Intervention and Data Collection

The feasibility study was conducted over a 4-week period in both facilities. All participants received a pair of study shoes equipped with integrated acceleration and pressure sensors. The system recorded gait and movement data continuously during daily activities whenever the shoes were worn. A brief introductory training session was provided to each participant to familiarize them with donning and doffing the shoes.

Weekly standardized walking tests (6‐12 min) were conducted under everyday conditions within the facilities. During these tests, the sensor system captured parameters such as step count, acceleration patterns, frequency and duration of movement, and ankle joint angle dynamics. Sensor data were wirelessly transferred to a secure analysis server during weekly on-site visits by the research team.

To complement real-world data collection, a series of controlled simulation procedures was conducted by the research team following predefined protocols (Multimedia Appendix 1). These simulations replicated characteristic agitation-related motor behaviors described in the *International Classification of Diseases, Tenth Revision* (ICD-10; eg, R45.1 “restlessness” and R46.3 “hyperactivity”), including restlessness in sitting and standing, repetitive nonlocomotor movements, and selected fall-like sequences. The simulated sequences served as reference data for exploratory model development and for examining the system’s ability to differentiate predefined movement signatures under controlled conditions.

The 2 data sources, simulation sequences and weekly walking tests, served distinct purposes. Simulation-derived data provided controlled reference patterns for algorithmic comparison, while longitudinal walking-test data allowed examination of continuous sensor function and variability across measurement occasions. Both datasets were processed using statistical and exploratory machine-learning procedures to support feasibility assessments regarding data capture, stability, and the detectability of movement signatures.

### Data Analysis

Data analysis was informed by the feasibility framework proposed by Orsmond and Cohn [21] and structured around three domains aligned with the guiding RQs: (1) process feasibility, (2) acceptability and perceived value, and (3) technical feasibility. Analyses examined how the system was used within everyday care routines, how residents and staff perceived its value, and under which practical conditions continuous movement-data acquisition was achievable in long-term care.

#### Process Feasibility (Recruitment, Study Procedures, and Organizational Integration)

This domain assessed the feasibility of conducting the study under routine institutional conditions. Three qualitative and descriptive data sources informed the analysis: nonparticipant behavioral observations (30-min sessions) during weekly walking tests, using a structured, time-stamped observation protocol. Informal staff feedback was collected continuously throughout the study period. Documentation of procedural deviations, including technical malfunctions, illness-related absences, and instances of nonuse of the shoe system. These data were analyzed descriptively to assess whether the study procedures were workable within daily routines, to identify facilitators and barriers to adherence, and to understand the organizational conditions influencing implementation feasibility.

#### Acceptability and Perceived Value (Residents and Staff)

To address RQ2, observational field notes and staff feedback were examined for indicators of residents’ and professional caregivers’ acceptance of the system. Content analysis focused on: observed user behaviors (eg, restlessness, engagement, and withdrawal), system-handling behaviors during everyday use, spontaneous verbal comments or reactions, perceptions of comfort, usefulness, and burden. The goal was to determine whether the system was tolerated or preferred by residents and how its perceived value shaped willingness to use it in daily care.

#### Technical Feasibility (Data Capture, System Functionality, and Exploratory Detection)

This domain (RQ3-RQ5) focused on the practicality and robustness of continuous sensor-based data acquisition under real-world conditions. Analyses included the following: completeness and quality of sensor data recorded during daily activities and weekly walking tests, type, frequency, and context of technical malfunctions; feasibility of capturing analyzable movement signals during naturally occurring restlessness or agitation; and exploratory examination of simulation sequences designed to elicit predefined agitation-related movement patterns. These analyses were descriptive and exploratory.

### Ethical Considerations

Data collection, storage, and analysis were conducted in compliance with data protection regulations. All participants received written and oral information about the study objectives, procedures, potential risks, and their rights and provided written informed consent prior to participation. Participation was voluntary, and participants could withdraw from the study at any time without providing a reason and without any negative consequences. No financial or other compensation was provided for participation. Personal data were pseudonymized, and the transmission and storage of sensor data were secured via a password-protected system with restricted access. The study was approved by the ethics committee of Eastern Switzerland (Req-2024-01190; EKOS24/163).

## Results

### Process Feasibility (Recruitment, Study Design, and Implementation): Recruitment Processes, Organizational Integration, and Day-to-Day Study Conduct (RQ1)

Recruitment was carried out across 2 long-term care facilities over a 12-week period. Each facility comprised 5 residential units, resulting in a recruitment pool of 10 units. Despite this coverage, only 6 residents (age range: 60‐81 y; 4 female and 2 male individuals) were ultimately enrolled as shown in Table 1. Screening was resource-intensive, as many residents did not meet the inclusion criteria due to advanced cognitive impairment, acute or fluctuating illness, limited mobility, or unavailability of legal consent. Recruitment required close and continuous coordination with nursing leadership to balance ethical considerations with practical feasibility.

**Table 1.** Participants within the study and intervention from weeks 1 to 4.

Characteristics				Week 1	Week 2	Week 3	Week 4	Notes
Participant	Sex	Age (y)	MMSE^a^ score					
T1	Female	80	23/30	✔	✔	✔	✔	—^b^
T2	Female	81	17/30	✔	✔	✔	✔	—
T3	Male	71	19/30	✔	✔	✔	✔	—
T4	Female	60	18/30	✔	✔	✔	✔	Went to hospital
T5	Female	79	17/30	✔	✔	✔	—	—
T6	Male	63	18/30	✔	—	—	—	Drop out

a MMSE: Mini-Mental State Examination.

b not applicable.

Participation during the study period showed considerable variability. Several residents missed individual measurement days due to scheduled medical appointments, illness episodes, or sedation. Only a small number of natural agitation episodes occurred. Care staff reported that some residents who typically display frequent agitation were pharmacologically sedated during data collection, which limited opportunities to capture agitation-related behaviors.

On the technical side, difficulties with the inductive charging process were frequent. Shoes needed to be placed correctly on the charging pad, and this step was occasionally missed or performed incorrectly, resulting in insufficient battery levels on scheduled data collection days. Rotating staff schedules and incomplete internal communication contributed to inconsistent handling, requiring repeated clarification by the research team and additional reminders at charging stations.

Organizationally, uneven information flow and shifting responsibilities within the multiprofessional team affected the regularity of system use. Acute health fluctuations, including fatigue, cognitive disorientation, hospital stays, or prescribed rest times, further limited opportunities for consistent measurement. Participant selection was also constrained by practical considerations such as shoe fit and cognitive ability to provide meaningful subjective feedback.

Participants also exhibited heterogeneous mobility levels, ranging from independent ambulation to short-distance walking with a rollator, often requiring nursing support, which influenced the practical organization of walking tests and sensor-based monitoring.

### Acceptability and Perceived Value in Everyday Care: User Perception and Acceptance (RQ2)

#### Acceptance Among Residents and Staff Perceptions

Resident acceptance varied considerably. The transition from commonly used open slippers to closed, form-fitting shoes was initially unfamiliar for several participants. Over time, many residents described an increased sense of stability while walking. Reports of feeling safer occurred even when the vibration feature was inactive, suggesting that perceptions were shaped mainly by the physical characteristics of the footwear. The vibration function elicited mixed reactions. Some residents did not notice the impulses or accepted them without comment, whereas others expressed irritation due to the unfamiliar sensation or perceived strength of the vibration.

Nursing staff reported perceived improvements in certain residents, such as calmer motor activity or more stable standing and walking. These impressions were often attributed to the system as a whole. Staff frequently did not differentiate between the mechanical support of the shoe and its technological features, which may reflect limited familiarity with sensor-based footwear systems.

#### Usage and Adherence

Adherence was generally satisfactory under study conditions, with residents and staff managing to incorporate the shoes into daily routines. Tolerance was high, as participants rarely expressed discomfort or rejected the footwear. However, perceived usefulness varied substantially among residents with dementia. While some described the system as helpful or engaging, others found no added value. This diversity highlights that acceptance does not necessarily translate into perceived personal benefit and that subjective relevance may be a critical factor for long-term adoption.

The presence of research personnel appeared to influence participant engagement. Many residents responded positively to the additional social interaction, suggesting that the research context may have contributed independently to increased activity levels or willingness to participate.

### Technical Feasibility (Data Capture and System Functionality)

#### RQ3 and RQ4: Real-World Data Capture Feasibility and Exploratory Assessment of Gait Characteristics Under Vibratory and Nonvibratory Conditions

Although naturally occurring agitation episodes were limited and heterogeneous, the study demonstrated that analyzable movement data could be obtained under real-world long-term care conditions. Preliminary inspection indicates that spatiotemporal gait parameters (eg, stride length and toe clearance) are technically extractable and may serve as candidate signals for subsequent agitation-related model development.

During daily use and weekly walking tests, the sensor system showed generally stable functionality whenever the shoes were worn and adequately charged. However, the frequent use of mobility aids (eg, rollators) altered walking patterns, introducing additional variability that future machine-learning models will need to account for, particularly in real-world care settings.

Vibration was not evaluated as an intervention but was examined exploratively to assess whether low-intensity tactile impulses produced measurable variations in gait characteristics. For each participant, several walking tests were conducted with vibration activated and deactivated in alternating order. During these tests, the following gait characteristics were calculated:

- Stride length: the stride length in meters
- Strike angle: the initial contact (strike) angle of the heel in degrees
- Heel clearance: the maximum heel clearance in meters
- Toe clearance: the minimum toe clearance in meters

Table 2 provides an overview of the observed ranges of variation and the proportion of comparisons showing statistically significant differences between vibration and nonvibration conditions.

**Table 2.** Overview of impact on gait characteristics^a^.

Participant	Stride length (m)	Strike angle (°)	Heel clearance (m)	Toe clearance (m)
T1	Range of impact: −0.06 to 0.12 Significant impact: 50%	Range of impact: −3.15 to 4.62 Significant impact: 25%	Range of impact: −0.02 to 0.02 Significant impact: 30%	Range of impact: −0.005 to 0.001 Significant impact: 10%
T2	Range of impact: −0.13 to 0.02 Significant impact: 29%	Range of impact: −7.15 to 6.10 Significant impact: 64%	Range of impact: −0.03 to 0.12 Significant impact: 43%	Range of impact: −0.004 to 0.005 Significant impact: 21%
T3	Range of impact: −0.09 to 0.17 Significant impact: 100%	Range of impact: −3.29 to 5.83 Significant impact: 66%	Range of impact: −0.01 to 0.02 Significant impact: 50%	Range of impact: −0.006 to 0.007 Significant impact: 33%
T4	Range of impact: −0.09 to 0.57 Significant impact: 75%	Range of impact: −3.66 to 7.73 Significant impact: 75%	Range of impact: −0.06 to 0.008 Significant impact: 25%	Range of impact: −0.029 to 0.006 Significant impact: 25%
T5	Range of impact: 0.03 to 0.09 Significant impact: 100%	Range of impact: −0.80 to 1.57 Significant impact: 50%	Range of impact: 0.007 to 0.016 Significant impact: 100%	Range of impact: −0.002 to 0.004 Significant impact: 75%

a Participant T6 was enrolled in the study but dropped out before data collection was completed. As no complete or analyzable data were available for this participant, T6 was not included in the table.

Across participants, no consistent or unidirectional effect of vibration was observed on the measured gait characteristics. Variability occurred in both directions (increases and decreases), and the proportion of statistically significant differences varied widely across parameters and individuals. Two exceptions were noted for participant T5, whose stride length and heel clearance showed consistently positive and statistically significant differences; however, these findings are based on only 1 measurement on each of 2 days and must therefore be interpreted with caution.

Overall, these exploratory results suggest that vibration-induced changes in gait, if present, are highly individualized and not robustly detectable with the current dataset.

#### RQ5: Detectability of Agitation Patterns in Controlled Settings

Controlled simulations were conducted to examine whether predefined agitation-related movement patterns could be captured and differentiated using the multisensor insole system. Four prototypical patterns based on ICD-10 agitation-related motor behaviors were repeatedly performed:

- Static sitting posture: feet firmly placed on the ground
- Seated toe-tapping: rapid, mainly vertical foot movements (tiptoeing)
- Seated foot-sliding: predominantly lateral foot movements (sliding)
- Standing toe-tapping: rapid, mainly vertical foot movements (tiptoeing) while standing

Each movement pattern was executed 40 times by 2 trained research staff members wearing the sensor-equipped shoes. All sessions were video-recorded for post hoc validation and cross-referencing with sensor data. Detailed descriptions of the testing procedures are provided in Multimedia Appendix 1.

**Figure 1.**
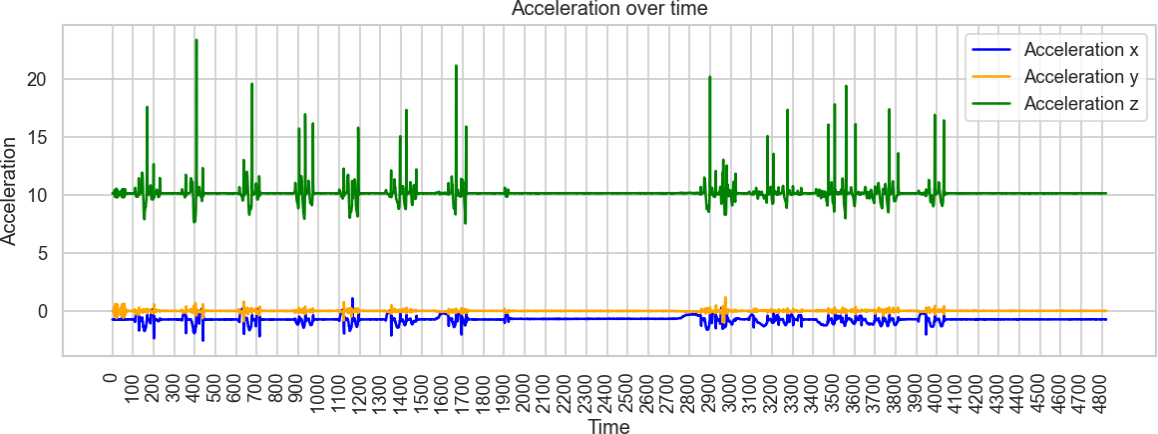
Example of acceleration measurements of seated toe-tapping.

Across these simulation trials, distinct acceleration signatures were observable for each movement type. The 3D accelerometer alone provided sufficient signal variation for preliminary classification, indicating that pressure sensors were not essential for differentiating the simulated patterns. Visual inspection of raw acceleration data confirmed that seated toe-tapping, seated foot-sliding, and standing toe-tapping produced characteristic oscillatory patterns in specific axes (Figures1^3^).

**Figure 2.**
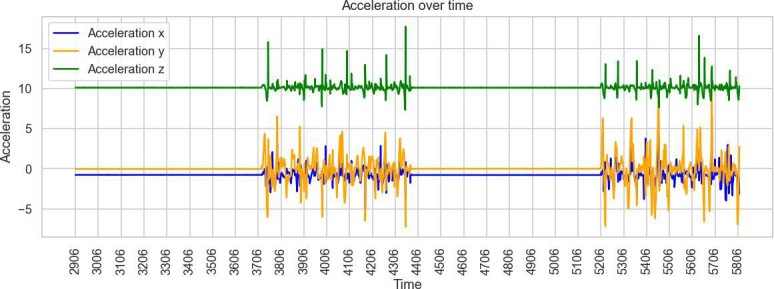
Example of acceleration measurements of seated foot-sliding.

**Figure 3.**
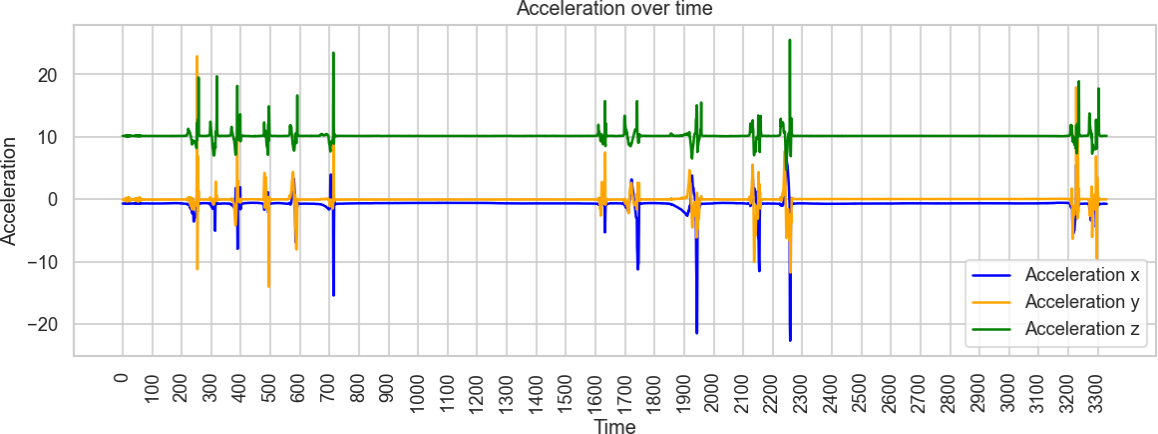
Example of acceleration measurements of standing toe-tapping.

Two exploratory machine-learning approaches were applied as follows:

1. Feature-based models (random forest, extreme gradient boosting, logistic regression, and simple neural network), using detrended and fast Fourier transform features.
2. A sequence-based hidden Markov model (HMM) using raw accelerometer data without preprocessing.

**Figure 4.**
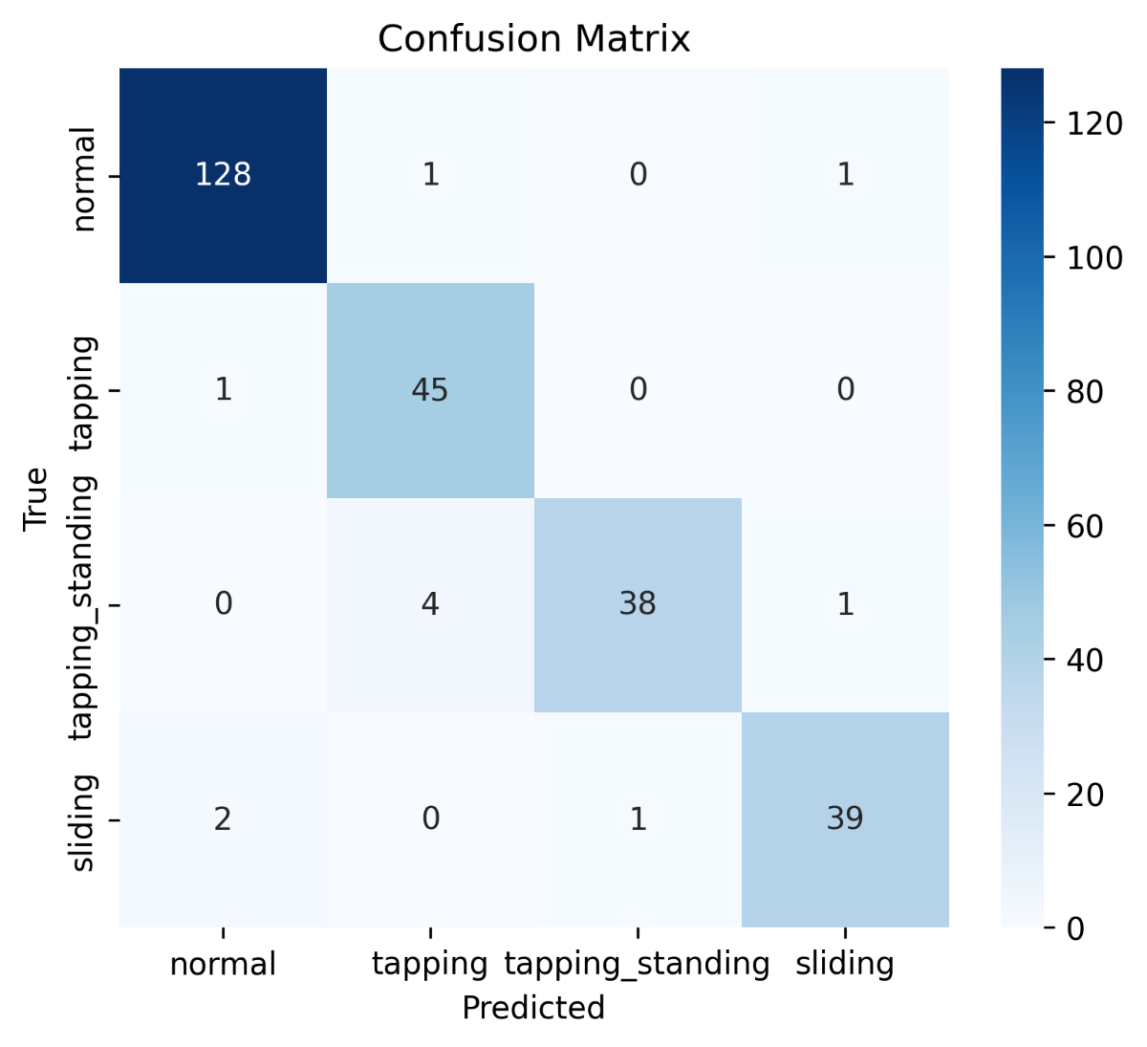
Confusion matrix of the random forest classifier.

The feature-based models classified nearly all test observations correctly, with the random forest, as illustrated in the confusion matrix (Figure 4), performing slightly better than the other classifiers (Table 3).

**Table 3.** Evaluation metrics of preprocessed classification models.

Pattern	Precision	Recall	*F*_1_-score
Static sitting posture	1.0	0.98	0.99
Seated foot-sliding	0.93	1.0	0.97
Seated toe-tapping	0.98	0.96	0.97
Standing toe-tapping	0.97	0.88	0.93

In contrast, the HMM approach showed lower precision and recall for the more dynamic patterns (particularly sliding and tapping while standing), although seated patterns were identified with higher accuracy (Table 4; Figures4  5). The learning curve (Figure 6) suggests that HMM performance is likely to improve with larger datasets.

**Table 4.** Evaluation metrics for the hidden Markov model classification.

Pattern	Precision	Recall	*F*_1_-score
Static sitting posture	1.0	0.82	0.90
Seated foot-sliding	0.74	1.0	0.85
Seated toe-tapping	0.98	0.98	0.98
Standing toe-tapping	0.76	0.93	0.84

**Figure 5.**
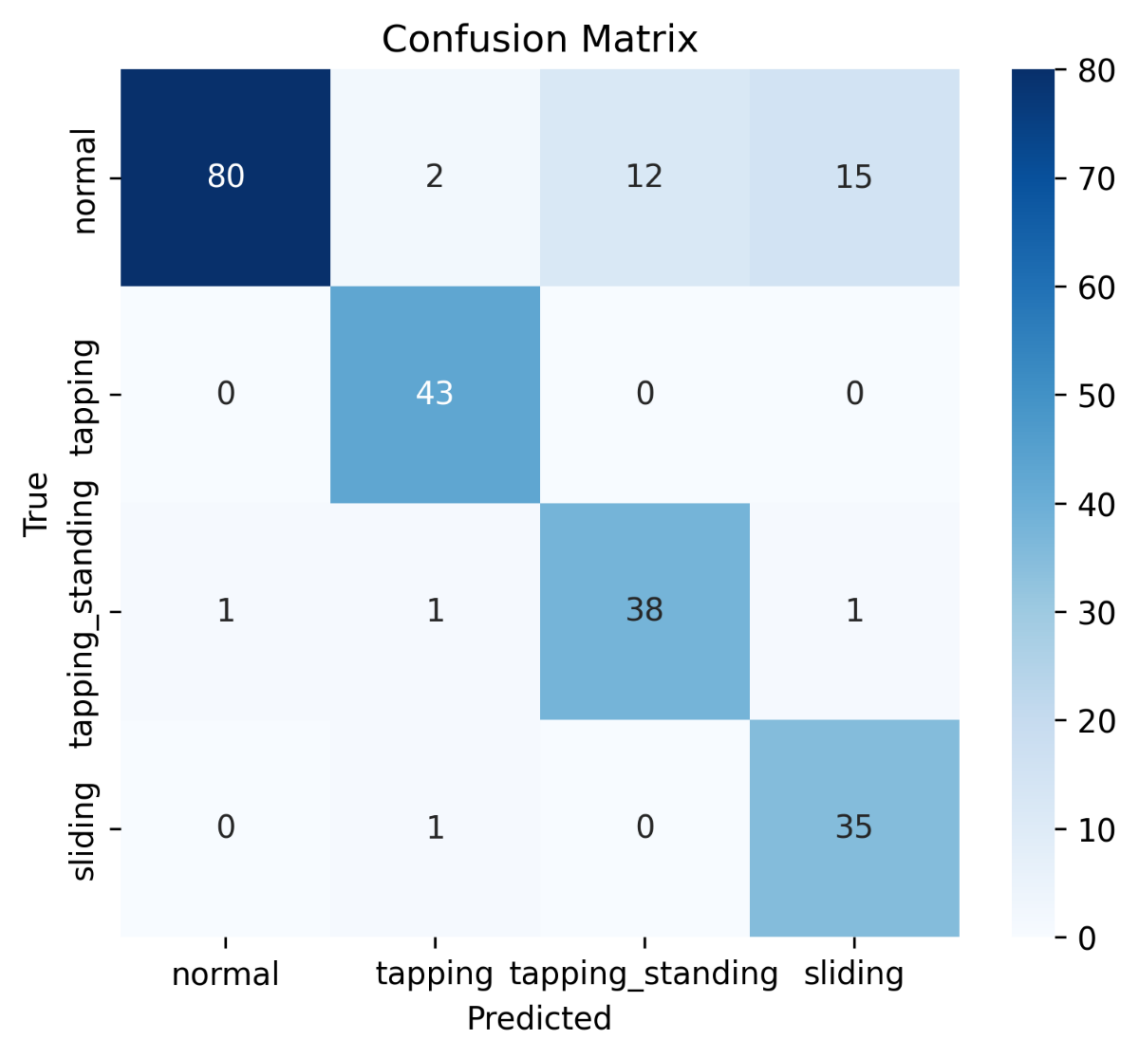
Confusion matrix of the hidden Markov model (HMM).

**Figure 6.**
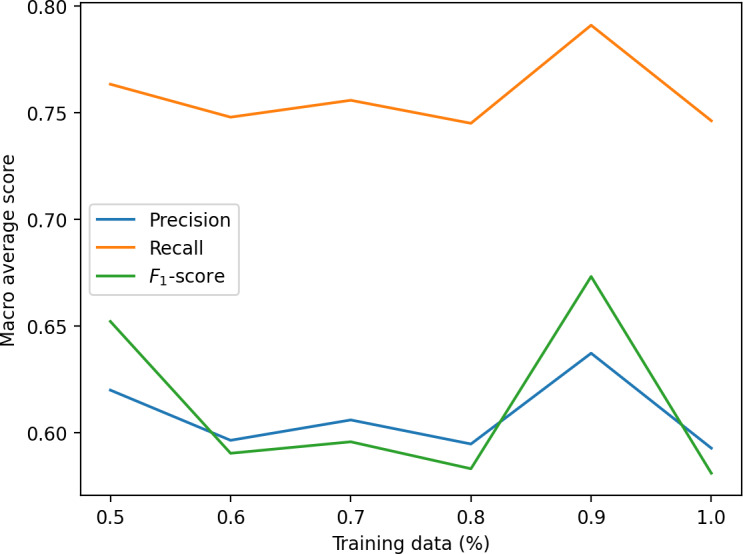
Learning curve for the hidden Markov model (HMM).

As a second, sequence-based approach, an HMM was used to identify the 4 states “normal,” “tapping,” “tapping_standing,” and “sliding.” The main advantage of HMM lies in the fact that there is no need for preprocessing and feature extraction; one can directly use the raw data from the 3D accelerometer. Accordingly, the need for processing power of a sensor system is lower than that for our first approach, which may enable a more resource-efficient implementation in future systems. As the confusion matrix in Figure 5 shows, the performance of the HMM algorithm is lower than that of the random forest.

The precision for “sliding” and “tapping_standing” is notably lower than that of random forest. Nevertheless, because HMMs are generative models that rely heavily on statistical estimates, they tend to benefit from larger datasets more than, for example, random forests, which increases the chances of improved performance with more training data. This idea is also supported by the calculated learning curve as shown in Figure 6.

These findings indicate that under controlled conditions, agitation-related motor patterns can be explored and differentiated using in-shoe accelerometer data, and that multimodal, feature-based models currently outperform raw-data sequence models for this dataset. These results should be considered preliminary, reflecting the limited sample and controlled environment.

## Discussion

### Integration of Findings Into Nursing Practice

This feasibility study provides initial insights into the acceptability and real-world practicality of sensor-based footwear for mobility and agitation-related movement analysis in institutional long-term care. Although the system demonstrated situational alignment with nursing needs, particularly regarding mobility support and movement-related data capture, its implementation was influenced far more by organizational and contextual conditions than by technical performance (eg, inconsistent charging routines). This is in line with implementation research showing that the integration of digital technologies in nursing care is strongly shaped by contextual and subjective factors such as nurse experience, structural conditions, and technical specifications which ultimately determine whether a technology can be embedded sustainably into everyday workflows [22-24]. The following operational constraints limited the extent to which feasibility could be interpreted in a broader implementation sense; communication gaps across rotating staff, limited availability of suitable shoe sizes, and resident-related fluctuations such as fatigue, illness, or episodic confusion repeatedly interfered with planned measurement sessions. These challenges affected protocol adherence and underscore the complexity of introducing new technological systems into routine care. Similar barriers, including inconsistent device handling, workflow disruptions, staffing variability, and fluctuating cognitive or health status, have been widely documented in implementation research on digital nursing technologies and wearables [10,12,13]. These studies collectively highlight that successful implementation depends not only on technological functionality but also on stable routines, device maintenance capacity, and the organizational readiness of care environments. Accordingly, feasibility should be interpreted with caution: although the system functioned under everyday conditions, achieving consistent and routine integration into care workflows would require additional organizational support, tailored training, and adaptations that account for the heterogeneity and unpredictability inherent in long-term care. Any future implementation would therefore require workflow-compatible procedures, clearly defined responsibilities, and organizational support structures capable of accommodating additional tasks such as charging devices, coordinating measurement schedules, and monitoring device use over time. Considerations related to resource allocation, including ongoing maintenance and staff training, further underscore the need to align technological solutions with the existing capacities and constraints of long-term care environments.

A clear discrepancy emerged between the intended target population and residents reachable under everyday institutional conditions. Sedation, health fluctuations, and inconsistent participation limited the capture of natural agitation. This underscores the tension between research design and long-term care realities, where dynamic health trajectories, risk management, and logistical constraints shape participation and data quality. These findings are consistent with prior work highlighting the need for pragmatic, context-sensitive approaches in complex care settings [25] and the inherent difficulty of attributing outcomes within multifaceted psychosocial and organizational nursing systems [26].

An additional aspect emerging from the study concerns the ethical tension between mobility promotion and safety-related practices. Sedation may reduce immediate risks associated with agitation but also restricts autonomy, mobility, and overall quality of life. While sensor-based monitoring could, in principle, contribute to earlier recognition of restlessness or instability, technologies must be implemented in a manner that supports person-centered care, respects residents’ comfort, and remains sensitive to ethical considerations. Their use should therefore be carefully calibrated to avoid reinforcing restrictive practices or adding burdens to already strained care routines.

Although this study was conducted in institutional long-term care settings, some insights may inform future adaptation of sensor-based footwear in home-based care. Portability across environments, independence from fixed-location infrastructure, and the potential to capture meaningful movement patterns in daily living contexts could offer advantages over stationary monitoring technologies. At the same time, the adoption of such systems in home care will depend on affordability, intuitive handling, and clearly perceived benefits for older adults and caregivers, factors consistently identified as essential determinants of technology acceptance in aging populations [27].

### Research at the Intersection of Standardization and Care Practice

This study illustrates the limitations of classical standardization in real-world long-term care. Methodologically rigorous and reproducible data collection often conflicts with the realities of everyday care [26,28]. These findings reinforce the broader insight that technological innovations cannot be meaningfully examined in isolation from the organizational environments in which they are situated.

Pragmatic exploratory studies such as this one play an important bridging role between technology development, nursing science, and day-to-day care delivery. They do not aim to produce classical randomized controlled trial evidence but instead generate practice-based insights that contribute to the growing body of “real-world evidence” [29]. In doing so, the study aligns with the goals of implementation science, which examines not only whether an intervention works but also how, for whom, and under what contextual conditions [30]. The findings highlight the central influence of nursing professionals and organizational culture on the meaningful use of sensor-based technologies in care environments [22]. By applying a context-sensitive and multiperspective approach, the study explores whether AI-supported sensor technology is not only technically functional but also ethically acceptable and organizationally compatible with the realities of nursing practice.

The study indicates that sensor-equipped footwear is generally acceptable for residents and feasible to use in principle. Although agitation detection appeared to have limited clinical relevance in this context, applications related to movement quality and fall-risk assessment seem more promising and may be achievable with simplified sensor configurations. Such configurations could support scalability and reduce resource requirements.

The findings also emphasize the importance of moving beyond sedation-centered behavioral management toward mobility-promoting and person-centered care models. Evidence from long-term care settings, particularly in the Netherlands and Belgium, suggests that nonpharmacological, activity-based approaches are associated with reduced agitation and lower reliance on psychotropic medication. In contrast, higher levels of staff distress have been associated with increased prescription of psychotropic drugs in residents with dementia [31-33]. Sustainable use of sensor-based systems would require workflow compatibility, adequate staff training, and supportive organizational structures. The project shows that a technology “drop-in” without structural embedding is unlikely to result in lasting adoption.

Residents generally accepted the device, and the technical functioning of the insole system could be demonstrated, yet several contextual barriers shaped feasibility. These included limited accessibility of the intended target group, frequent pharmacological sedation, acute health fluctuations, inconsistent charging, communication gaps across shifts, and practical issues such as shoe fit. Together, these observations offer an empirical foundation for refining implementation strategies and designing larger studies aimed at evaluating feasibility and early effectiveness under more controlled operational conditions.

Beyond implications for study feasibility, these findings also raise questions about the practical value of sensor-based agitation detection in everyday long-term care. In settings where agitation is predominantly managed through pharmacological sedation or restrictive practices, residents who might benefit most from continuous monitoring are often those for whom detectable agitation-related behaviors are least observable. Under such conditions, sensor-based agitation detection may have limited immediate clinical use. However, the absence of detectable agitation should not be interpreted as the absence of underlying distress. From a longer-term perspective, sensor-based movement monitoring may still provide value by making care practices more visible, supporting reflection on sedation use, and enabling the evaluation of alternative, mobility-oriented approaches to managing behavioral symptoms. The findings, therefore, suggest that the added value of agitation detection technologies is strongly context-dependent and closely linked to institutional care cultures and management strategies.

### Limitations

This feasibility study has several limitations that should be considered when interpreting the findings. The sample size was very small and shaped by substantial recruitment barriers, including prevalent sedation practices and fluctuating health status, which restricted the pool of eligible participants. These constraints limited the diversity and representativeness of the sample and affected the consistency of participation across measurement days.

Natural agitation episodes occurred only rarely during the study period, which restricted the opportunity to observe spontaneous agitation-related behavior under real-world conditions. Although controlled simulations served as a methodological proxy to characterize agitation-related movement patterns, they cannot fully substitute for naturalistic observations and should therefore be interpreted as exploratory.

The machine-learning analyses relied on a restricted and highly standardized dataset derived largely from controlled testing, which increases the risk of overfitting and limits generalizability. Larger datasets with more heterogeneous patterns will be necessary to develop robust detection models.

Furthermore, inconsistent charging routines, workflow interruptions, and variable communication across shifts affected protocol adherence and introduced procedural variability that may have influenced data completeness and system performance.

These limitations are typical for early-stage feasibility studies in complex care environments. They nonetheless highlight important methodological considerations for future research, including the need for expanded samples, improved charging and communication routines, and study designs that more effectively accommodate the unpredictability of long-term care.

### Conclusions

This feasibility study demonstrates that sensor-based footwear is generally acceptable to residents and has the potential to be technically feasible under everyday conditions in long-term care. Real-world contextual factors, particularly pharmacological sedation, fluctuating health status, and organizational constraints (eg, inconsistent charging routines) significantly hinder the system’s everyday usability. These constraints delineate the feasibility boundaries of sensor-based agitation monitoring and clarify why certain target outcomes could not be meaningfully assessed in this setting.

Our findings provide essential early insights into the contextual determinants that shape the use of sensor-based technologies in institutional care. They identify the technological refinements, procedural adjustments, and organizational prerequisites that must be addressed before larger-scale evaluations can be conducted. By revealing how feasibility is conditioned by institutional routines, staffing structures, and resident characteristics, the study lays important groundwork for subsequent research and supports the development of digital innovations that are aligned with everyday nursing practice. Ultimately, such technologies have the potential to promote mobility, autonomy, and well-being provided they are adapted to the realities of long-term care and embedded in supportive organizational frameworks.

## Supplementary material

10.2196/83133 Multimedia Appendix 1 Simulation protocol for algorithm validation.

## Abbreviations

AI: artificial intelligence
HMM: hidden Markov model
ICD-10: *International Classification of Diseases, Tenth Revision*
RQ: research question
