# Supplementary material for: Insights on Recruitment, Implementation, and Movement Pattern Detection by Exploring the Feasibility of Sensor-Based Insole Technology in Long-Term Care: Mixed Methods Feasibility Study
Source: JMIR Form Res. 2026 Feb 18;10:e83133. doi: 10.2196/83133 (PMC12916087; doi:10.2196/83133)
Supplement: Multimedia Appendix 1 [file formative-v10-e83133-s001.docx]

# Supplement 1: Simulation Protocol for Algorithm Validation

To validate the classification algorithms for agitation-related motor activity, a structured series of movement simulations was conducted under controlled conditions during the feasibility phase. All simulations were designed to replicate clinically relevant agitation patterns as defined by the ICD-10 (e.g., R45.1 "restlessness," R46.3 "hyperactivity") as well as trough field observations and were informed by empirical observations in long-term care settings.

Each movement pattern was performed 40 times by two trained research staff members wearing the sensor-equipped shoes. All sessions were video-recorded for post-hoc validation and cross-referencing with sensor data. Simulations included both stationary and spatially dynamic behaviors, as well as a fall scenario, as detailed below:

Case 1: “Normal sitting posture”
Simulated condition: The participant is seated with both feet resting flat and motionless on the ground.
Purpose: Serves as a baseline to assess sensor behavior under stable conditions.

Case 2: “Seated toe-tapping”
Simulated condition: The participant is seated and performs repetitive, vertical foot movements without spatial displacement (e.g., rhythmic toe lifts).
Purpose: Simulates subtle restlessness or tremor-like behavior in a seated posture.

Case 3: “Seated foot-sliding”
Simulated condition: The participant is seated and moves the feet horizontally along the floor in a repetitive, lateral motion.
Purpose: Mimics horizontal foot shifts or sliding behavior, resembling motor restlessness.

Case 4: “Standing toe-tapping”
Simulated condition: The participant is in an upright standing position and performs repetitive up-and-down foot motions without displacement.
Purpose: Represents restlessness or agitation while standing, relevant for detecting early signs of instability or nervous pacing.

All simulations were conducted in a standardized environment to ensure consistency across trials. These structured cases provided a labeled dataset for algorithm training and preliminary validation under semi-controlled real-world conditions.
